# Band engineering in a van der Waals heterostructure using a 2D polar material and a capping layer

**DOI:** 10.1038/srep27986

**Published:** 2016-06-15

**Authors:** Sung Beom Cho, Yong-Chae Chung

**Affiliations:** 1 Department of Materials Science and Engineering, Hanyang University, Seoul 133-791, Korea.

## Abstract

Van der Waals (vdW) heterostructures are expected to play a key role in next-generation electronic and optoelectronic devices. In this study, the band alignment of a vdW heterostructure with 2D polar materials was studied using first-principles calculations. As a model case study, single-sided fluorographene (a 2D polar material) on insulating (*h*-BN) and metallic (graphite) substrates was investigated to understand the band alignment behavior of polar materials. Single-sided fluorographene was found to have a potential difference along the out-of-plane direction. This potential difference provided as built-in potential at the interface, which shift the band alignment between *h*-BN and graphite. The interface characteristics were highly dependent on the interface terminations because of this built-in potential. Interestingly, this band alignment can be modified with a capping layer of graphene or BN because the capping layer triggered electronic reconstruction near the interface. This is because the bonding nature is not covalent, but van der Waals, which made it possible to avoid Fermi-level pinning at the interface. The results of this study showed that diverse types of band alignment can be achieved using polar materials and an appropriate capping layer.

Van der Waals (vdW) heterostructures are considered to be a key component of next-generation electronics and optoelectronic devices^1^. These heterostructures are composed of a combination of layered materials and two dimensional (2D) materials^2^. These materials have advantages for designing interface characteristics as compared to traditional heterostructures like oxide and III–V semiconductors. Because the bonding at the interface is mainly vdW, these heterostructures do not have the problems encountered in traditional heterostructures, which are mainly due to dangling bonds at the interface. For traditional heterostructures, various reconstructions, including defect generation and atomic intermixing, must occur to compensate for dangling bonds at the interface^3^,^4^. However, these reconstructions degrade the device characteristics. There are no dangling bonds in vdW heterostructures, therefore the interface is atomistically sharp^5^. As such, the properties of each material at the interface are preserved without any degradation.

Another fascinating feature of vdW heterostructures is the diversity of material combinations available for heterostructure design. For traditional heterostructures, the lattice parameter of each material must be matched within a tolerance in order to minimize the number of dangling bonds^6^,^7^. However, when materials are bonded with vdW forces, the lattice parameter constraint is meaningless because there are no dangling bonds^8^,^9^. In principle, the desired heterostructures can be achieved by stacking materials using transfer techniques^10^,^11^. The desired functionality is achieved by selecting appropriate materials and controlling their interactions. Diverse heterostructures have been fabricated for various electronic and photonic device applications using this method^11^,^12^,^13^,^14^,^15^.

One of the crucial factors for determining device characteristics is the band alignment at the interface. Various band engineering methods have been extensively invenstigated in order to achieve the desired band alignment^16^,^17^. Among these, the use of polar material is one of the most efficient methods for band engineering^18^. These polar materials have an intrinsic electric field that induces a built-in potential at the interface^19^ and this built-in potential modulates the band alignment. This approach has been widely adopted in traditional heterostructures for the purpose of band engineering. An example of this is an interface with a polar oxide, LaAlO_3_, and non-polar oxide, SrTiO_3_^20^. The LaAlO_3_/SrTiO_3_ interface showed staggered gap when the thickness of LaAlO_3_ was less than 3 unit cells. As the thickness of LaAlO_3_ increased, the size of the staggered gap decreased due to the built-in potential induced by the polarity of LaAlO_3_. An insulator-metal transition occurs when the thickness of LaAlO_3_ is above a critical thickness^21^. Previous investigators showed that band engineering with polar materials can be more finely tuned when a capping layer (CL) is employed^22^. This approach allows fine tuning beyond the digital control of the thickness of the polar materials. An insulator-metal transition was observed below the critical thickness for LaAlO_3_/SrTiO_3_ with a SrTiO_3_ CL^23^. Depending upon the choice of the metallic CL, the spin polarisation or an additional carrier injection can be achieved at the interface^24^,^25^. This is because the CL provides an electrostatic boundary condition for the polar materials, which changes the properties.

Despite numerous studies^26^, there is lack of understanding of how polar heterojunctions influence the band alignment. This is because the complex secondary effects make it difficult to isolate the effects of polar materials, particularly in covalently bound heterostructures. Numerous variables can affect the band alignment due to covalent bonding at the heterojunction. The induced energy level of a covalent bond can result in Fermi-level pinning, which influences band alignment^27^. Also, band bending and charge transfer occurs, in order to meet the charge state at the interface, and these also influence the band alignment^17^. Furthermore, atomistic defects near the interface can hinder the effects of a polar material by pinning the Fermi level^28^.

Fortunately, those problems can be avoided in a vdW heterostructure because it does not rely on covalent bonds. For this reason, a pure response to the polarity might be observable when polar materials are employed in vdW heterostructures. Recently, it has been reported that 2D polar materials like single-sided fluorographene (C_4_F) can modulate the band alignment and other functional characteristics^29^,^30^. For instance, the C_4_F can be used modulate the band alignment in the BN/C_4_F supercells and can induce an insulator-metal transition by controlling the thickness of each component. Though polarity-triggered band engineering at vdw interface has been reported, the response to polarity at the interface has not been systematically investigated. Considering that the band alignment of polar materials depends on the boundary conditions, it is necessary to investigate the band alignment for various the boundary conditions. For these reasons, investigating vdw heterostructure with polar materials can not only provide fundamental understanding of the interface physics of the polarity, but also guidelines for band engineering.

In this study, we investigated the band alignment of vdW heterostructures using 2D polar materials. We chose C_4_F as a representative 2D polar material. The effect of polarity on the band alignment was studied at the heterostructure, and the effect of CL on the band alignment was also invenstigated. Band alignment was expected to respond differently with polar materials because the presence of a CL changes the electrostatic boundary from vacuum to metallic or dielectric. To investigate this effect, monolayer graphene and BN were adopted as a representative metallic and insulating CLs, respectively. We found that the band modulation caused by polarity depended on the potential step of C_4_F, and this potential step can be tuned with a boundary condition change using a CL.

## Results

**Figure 1:**
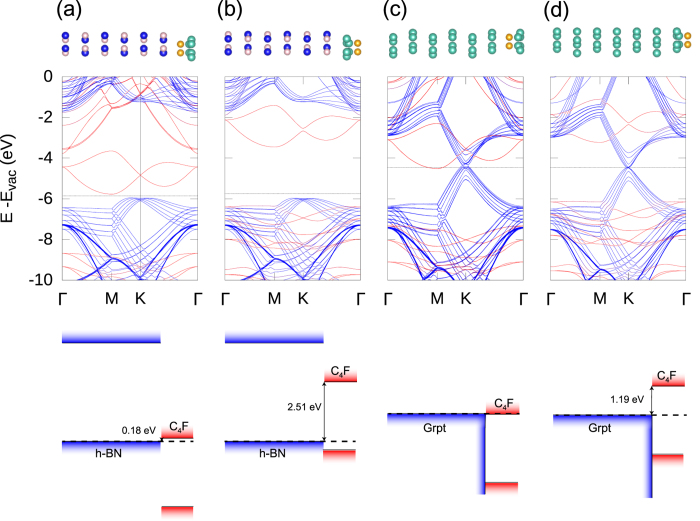
Atomistic configurations, band structures and corresponding band diagrams for C_4_F on *h*-BN and graphite substrates. Blue, pink, orange, and cyan balls represent nitrogen, boron, fluorine, and carbon atoms, respectively. The band of the substrate is colored with blue and that of C_4_F is red. The Fermi-level of the system is plotted with a dotted-line. The band alignment highly depends on the termination because of the intrinsic potential difference in the polar material, C_4_F.

To investigate the band alignment between single-sided fluorographene and the substrates, we plotted the band structure and corresponding band diagram in Fig. 1. In the band structure, the vacuum level of the substrate side was set to zero on the energy scale. This side of the vacuum level provided a reference value for different systems because the substrate was thick enough to avoid interfering with the interface. Using this reference value, we compared all possible interface terminations of the C_4_F, C-side and F-side contact with BN and graphite substrates. As shown in the band structures, the band alignment at the interface is highly dependent on the termination. The F-side contact of C_4_F and BN (C_4_F/*h*-BN) had a narrow gap of 0.18 eV. On the other hand, when the C-side of C_4_F was in contacted with BN (FC_4_/*h*-BN), a staggered gap of 2.51 eV formed with a conduction band minimum (CBM) of C_4_F and a valence band maximum (VBM) of *h*-BN. Considering the bandgap is underestimated when using DFT with PBE functional, we also calculated the staggered gap using a hybrid functional calculation. The 1.45 eV and 3.81 eV were obtained for the staggered gap of C_4_F/*h*-BN and FC_4_F/*h*-BN interfaces, respectively. The obtained values showed that the PBE functional still captured the essential trends of band alignment including the band shift between the two terminations. This termination dependency was also observed at the interface between C_4_F and graphite. The Ohmic contact was formed on the F-side contact (C_4_F/Grpt), while the C-side contact (FC_4_/Grpt) showed a Schottky nature.

As shown in the plot, the band alignments at the interface were mainly determined by the C_4_F band. While the position of the band of the substrates was kept constant, the band of C_4_F shifted depending on the termination. This shift is because of the dipole moment of C_4_F. A strong dipole moment is generated in C_4_F due to the asymmetric electronegativity between carbon and fluorine^31^. This dipole moment along the *z* direction was calculated using the following equation.


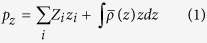


Here, Z_*i*_ is the charge of core of ions *i*, and z_*i*_ is the position of the ion along the *z* side. The planar-averaged electron density of the slab along the z-direction is 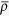. The calculated dipole moment for single layer C_4_F along the z-side is 0.270 eÅ. This dipole moment was quite large value and corresponds to the 7 layers of LaAlO_3_^21^. This dipole moment induced an electrostatic potential difference between the C-side and F-side of C_4_F. The electrostatic potential difference was evaluated using the Helmholtz’ equation.


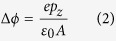


Here, *ϕ* is the electrostatic potential, *ε*_0_ is the dielectric constant, and *A* is the area of the supercell. The potential difference was 2.33 eV, which corresponds to the amount of the band shift between the C-side contact and the F-side contact in C_4_F on the BN substrate. Furthermore, this large potential difference is sufficient to convert the Schottky contact on the C-side in FC_4_/Grpt to an Ohmic contact on the F-side in C_4_F/Grpt because the Schottky barrier is only 1.19 eV.

**Figure 2:**
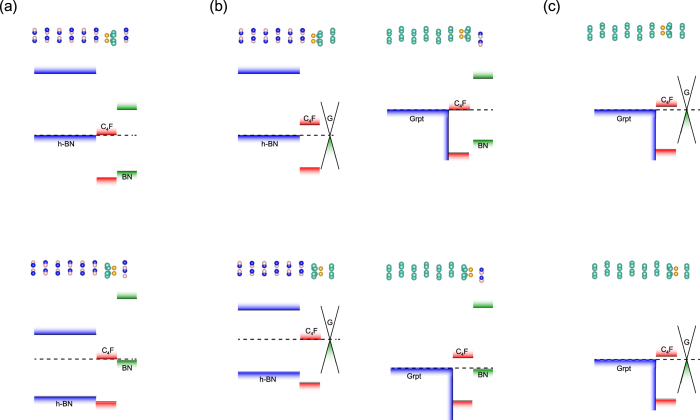
Band diagram of the CL, C_4_F, and substrates. Depending on the electrostatic boundary condition for C_4_F, the interface type is classified into one of three categories. (**a**) type-A for two insulating substrates, (**b**) type-B for one metallic and one insulating, (**c**) type-C for both metallic boundary conditions.

Considering the side-dependency of the band alignment of C_4_F, we expected that the band alignment with the next overlayer, CL, will be affected by the polarity of C_4_F. Thus, the effect of CL on the band alignment was also investigated. The BN and graphene layers were selected as representative CLs and were deposited onto the C_4_F on the BN substrate and graphite substrate. A schematic band diagram of the interfaces is provided in Fig. 2. Interestingly, the behavior of the band alignment between the substrate/C_4_F/CL highly depends on the combination of the CL and substrates. This is because the substrate and CL provide different electrostatic boundary conditions for C_4_F. For this reason, we classified these interfaces into three categories according to their electrostatic boundary conditions: type-A has two insulators, type-B has one insulator and one metal, and type-C has two metals.

**Figure 3:**
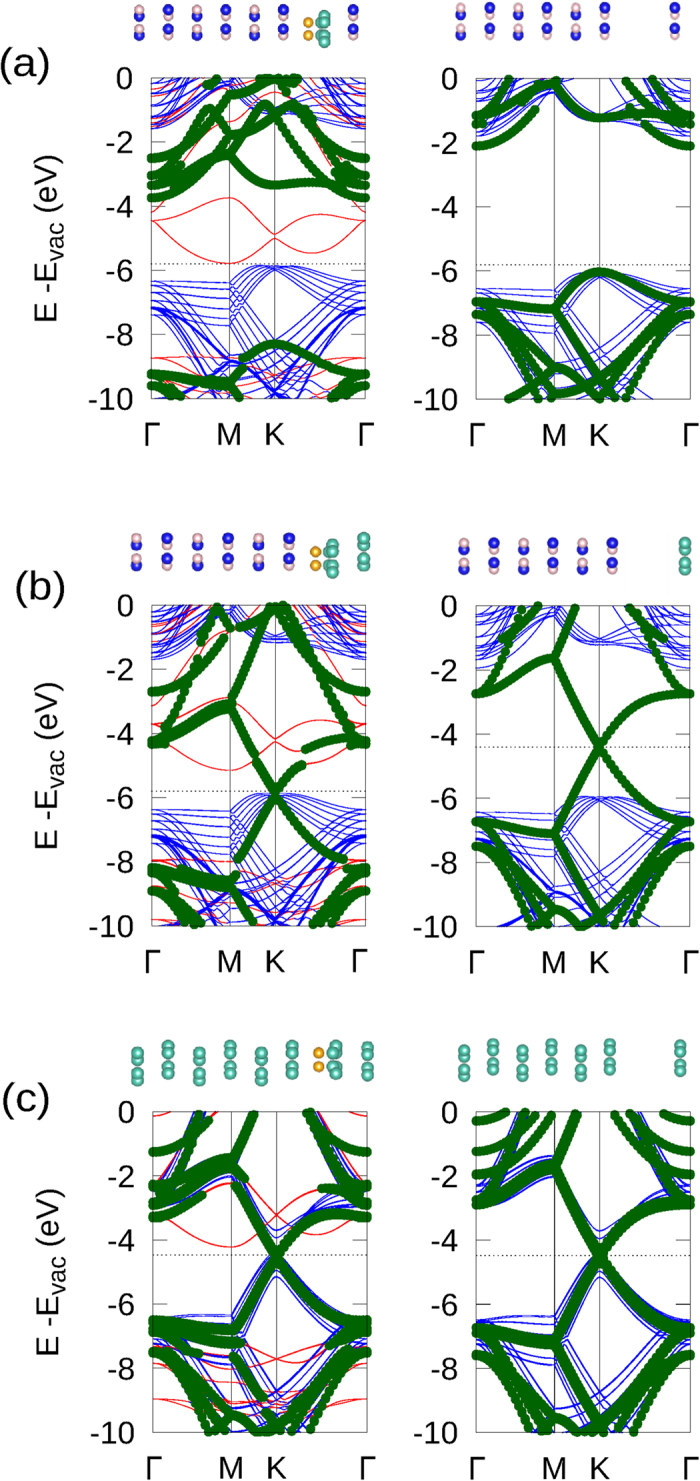
The band structure of CL/C_4_F/substrates and CL/substrates for each representative case. For CL/substrates, the interspace was fixed to at least 5 Å to observe the alignment to vacuum and avoid the secondary effects. The blue and red line show the band structure of the substrate and C_4_F, respectively. The green dots represent the band of the CL. (**a**) The band structure of type-A and the band shift corresponds to the intrinsic potential difference of C_4_F. (**b**) The band structure of type-B and the band shift of CL is less than the intrinsic potential difference of C_4_F. (**c**) The band structure of type-C, which does not show a band shift in the CL.

The band alignment between the substrate and the CL was determined by the potential difference between the sides of C_4_F. To observe this potential difference, we compared the band diagrams of the two systems, the CL/C_4_F/substrate and the CL/substrate. In the CL/substrate, the interlayer spacing was fixed to at least 5 Å, in order to observe the alignment reference to vacuum and exclude the interface interaction between the substrate and the CL. Here, we calculated the band structure by optimising the system when the C_4_F layer was removed. This distance of 5 Å was sufficient to avoid the interactions between the layers, allowing us to observe the band alignment with respect to the vacuum level^18^,^32^. Figure 3 shows a comparison of the representative case for each alignment type. The BN on C_4_F/*h*-BN (BN/C_4_F/*h*-BN), the graphene on C_4_F/*h*-BN (G/C_4_F/*h*-BN), and graphene on C_4_F/Grpt (G/C_4_F/Grpt) were chosen as the representative cases for type-A, type-B, and type-C, respectively. In type-A, the band of the CL shifted by 2.33 eV in the CL/C_4_F/substrate as compared to CL/substrate. This amount of shift exactly matched the intrinsic potential difference of C_4_F. This means that the band alignment of type-A was determined by the intrinsic potential difference of C_4_F. On the other hand, the shifts in type-B and type-C did not correspond to the intrinsic potential difference of C_4_F. In type-B, the amount of the shift was 1.49 eV, which was a remarkably lower than the intrinsic potential difference of C_4_F, 2.33 eV. The same tendency was observed in type-B. The shifts were 1.41 and 1.30 eV in BN/FC_4_/*h*-BN and BN/FC_4_/Grpt, respectively (see supporting information). All of these values were less than the intrinsic potential difference, 2.33 eV. Interestingly, CL was aligned with the same energy leveln type-C, which means that there was no potential shift arises in type-C. These results for type-B and type-C indicate that the band alignment was not determined merely by the intrinsic potential difference of C_4_F.

To understand this kind of different behavior, we also calculated the electron redistribution function as follows.


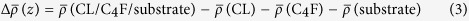


**Figure 4:**
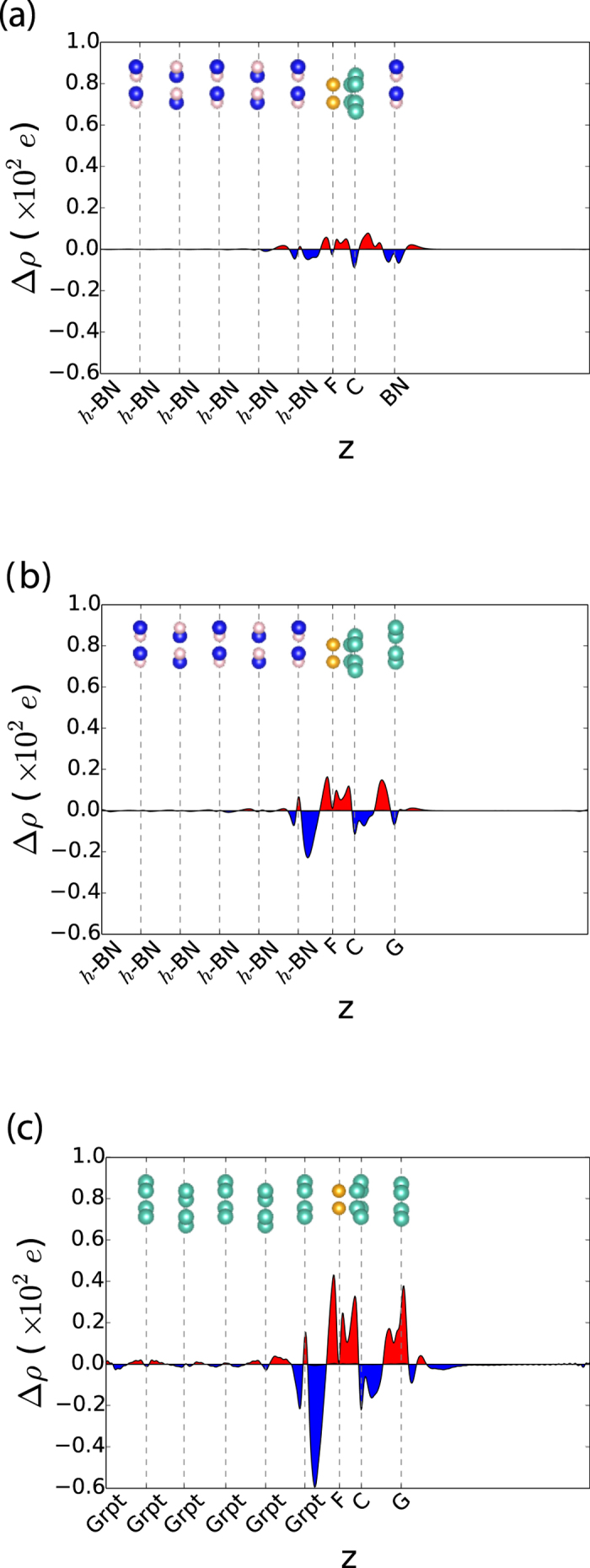
Electron redistribution function for the representative cases for (**a**) type-A, (**b**) type-B, and (**c**) type-C. The accumulation region is red, and the depletion region is blue. The redistribution generates an electric field with a direction that canceled out the intrinsic electric field of C_4_F.

The results for the representative cases of type-A, B, and C are plotted in Fig. 4. Though the magnitude of the redistribution is different for each type, the patterns in the accumulation and depletion regions of the redistribution were quite similar. Electron depletion was observed on the substrate side of the space between the C_4_F and the substrate, while the electrons are accumulated on the C_4_F side. On the other hand, the electrons between the C_4_F and CL interface accumulated on the C_4_F side, but depleted on the CL side. This kind of redistribution can be interpreted as spatial polarisation or charge transfer. This phenomenon is apparent in the band structure in Fig. 3. If an electron was transferred to C_4_F, it should occupy the conduction band minimum (CBM) of C_4_F. However, as shown in Fig. 3, the conduction band of C_4_F was empty. Therefore, the redistribution can be interpreted as spatial polarisation rather than charge transfer. This spatial polarisation also induced a dipole moment, and the direction of this polarisation was opposite to the intrinsic dipole moment of C_4_F. This means that the intrinsic dipole moment of C_4_F was screened by spatial polarisation. Considering the band shifts of CL, the intrinsic dipole of C_4_F was partially screened in type-B and completely screened in type-C.

The magnitude of the screening depended on whether the boundary conditions contained a metal or not. Here, graphene or graphite screened the dipole moment of C_4_F. One interesting feature was that the spatial polarisation was not localized at the region near the metal. As shown in Fig. 4b, redistribution in the region of C_4_F/*h*-BN was also observed. Originally, when the CL was absent, the magnitude of the redistribution was very similar to that of type-A in Fig. 4a (see supporting information). However, when metallic CL is added, the electron redistribution increased substantially, as shown in Fig. 4b. The redistribution between the substrate and C_4_F changed the barrier height between C_4_F and *h*-BN. Before the graphene CL was deposited, the staggered gaps between C_4_F and *h*-BN is 0.18 eV and 2.51 eV for C_4_F/BN and FC_4_/BN, respectively, as shown in Fig. 1. When the graphene was used as the CL for C_4_F on *h*-BN substrate, the staggered gap of C_4_F/*h*-BN increased from 0.18 eV to 0.65 eV, and the gap of FC_4_/*h*-BN is decreased from 2.51 eV to 2.21 eV, as shown in Fig. 2. Furthermore, by depositing CL, the junction properties between the graphite substrate and C_4_F were controlled by depositing CL. The C_4_F/Grpt interface junction was the Ohmic in nature, as shown in Fig. 1c. This Ohmic nature was converted to a Schottky contact after the graphene cap was applied, as shown in Fig. 2c. The p-type Schottky barrier of 1.19 eV in FC_4_/Grpt showed a remarkable reduction to 0.24 eV after the capping with graphene. This change in the interface property was due to electron redistribution where the intrinsic dipole of C_4_F was screened. On the other hand, the type-A interface, which had a negligible screening effect by capping, preserved its own barrier height. This means that the barrier height was modulated due to the screening effect by the metallic CL.

The origin of this type of band modulation by the CL was also observed in an oxide interface. A more significant band modulation is observed at the LaAlO_3_/SrTiO_3_ interface when CL was metallic rather than insulating. However, the mechanism for the band modulation in the oxide heterostructure was different from that of a vdw heterostructure. In the oxide heterostructure, the Fermi-level pinning induced by the metallic CL is the key factor that determines the band alignment^25^,^33^. Due to the pinning, the band alignment between metal and SrTiO_3_ in metal/LaAlO_3_/SrTiO_3_ was very similar to that of metal/SrTiO_3_^24^. Fermi-level pinning can trigger a charge transfer resulting in occupation of the conduction band of SrTiO_3_. This electron in the conduction band of SrTiO_3_ screens the intrinsic electric field of LaAlO_3_. On the other hand, such remarkable Fermi-level pinning or charge transfer has not been observed in vdW heterostructures. Instead, the response to the polarity in vdW heterostructure was mainly derived from the electron redistribution in the interface region. This redistribution screens the dipole moment of the C_4_F, and thus modulates the band alignment. This change implies that types of bonding in a heterostructure is an important factor in determining the response to the polarity at the interface.

## Conclusion

In summary, we investigated band alignment in vdW heterostructures with polar materials and a CL. The band alignment was found to be highly dependent on the intrinsic electric field of the polar materials, and this can be additionally modified with a CL. The staggered gap of C_4_F/BN can be controlled by depositing a CL on the polar materials. Furthermore, even Ohmic-Schottky transition in C_4_F/Grpt can be triggered by a CL. This wide range of tunability results from the polarity screening of the CL due to the different responses to the polarity in vdW heterostructures. Because of the absence of covalent bonding in the heterostructure, the response mainly spatial involves polarisation at the interface region. This demonstrates that band engineering with polar materials and a CL is highly effective for achieving a desired device deisgn.

## Methods

First-principle calculation were performed using the Vienna ab-initio simulation package (VASP)^34^. The interactions between the ions and electrons were described using the projector-augmented-wave method with a 500 eV cutoff energy for the plane wave basis set^35^. The kinetic energy cutoff in the construction of the plane wave basis was set to 500 eV. To include vdw interaction in the exchange-correlation energy, the optB88-vdW functional was chosen for the exchange part and the Perdew-Burke-Ernzerhof (PBE) functional was chosen for the correlations part. All of the self-consistent loops were iterated until the total energy difference of the systems between the adjacent iterating steps was less than 10^−5^ eV. The *h*-BN and graphite substrate was described using 6 layers of graphene and BN. The thickness of the six layers was at least 1.5 nm for both cases, and this prevented the surface effects of the substrate from influencing the interface interactions. In order to avoid dipole-dipole interaction between the periodic images, a vacuum level of at least 15 Å was inserted and a dipole correction was applied. All the atoms were fully relaxed until the maximum total Hellmann-Feynmann force was less than 0.01 eV/Å. The calculated lattice parameter of C_4_F was 4.92 Å, which agrees well with previous theoretical studies^29^,^31^. The periodicity of the interface is based on 1 unit cell of C_4_F and (2 × 2) cells of graphene and BN. The modeled interface was calculated using 9 × 9 × 1 sampling with the Monkhorst-Pack method in the Brillouin-zone^36^. The lattice mismatch was below 1% in this periodicity because the lattice constant of both materials was 2.46 Å.

For calculated band structure, each of bands are projected onto the spherical harmonics of each atom. Based on this projection, we decided which bands belonged to which component using the following equation.


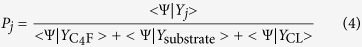


Here, the P is the value of projection of component j, Ψ is the wave function, and Y is the spherical harmonics of each atom in component. We determined the components of the bands based on the largest value of P_*j*_.

## Additional Information

**How to cite this article**: Cho, S. B. and Chung, Y.-C. Band engineering in a van der Waals heterostructure using a 2D polar material and a capping layer. *Sci. Rep*. **6**, 27986; doi: 10.1038/srep27986 (2016).

## Supplementary Material

Supplementary Information
