# Supplementary material for: Band engineering in a van der Waals heterostructure using a 2D polar material and a capping layer
Source: Sci Rep. 2016 Jun 15;6:27986. doi: 10.1038/srep27986 (PMC4908394; doi:10.1038/srep27986)
Supplement: Supplementary Information [file srep27986-s1.pdf]

Supporting information for: Band engineering in a van der Waals heterostructure using a 2D polar material and a capping layer

Sung Beom Cho and Yong-Chae Chung*

Department of Materials Science and Engineering, Hanyang University, Seoul 133-791

E-mail: yongchae@hanyang.ac.kr

*To whom correspondence should be addressed

Table S1: Stacking configuration of single-sided fluorographene with CL and substrates.

	Atomic structure	Stacking pattern	Interlayer distance w.r.t. CL (Å)	Interlayer distance w.r.t. substrate (Å)
C_4F/BN	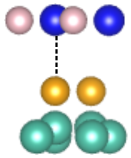	A-B	2.81	2.81
FC_4/BN	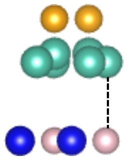	A-B	3.27	3.28
C_4F/G	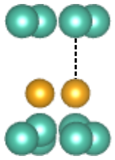	A-A	2.89	2.85
FC_4/G	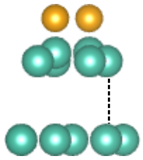	A-B	3.31	3.31

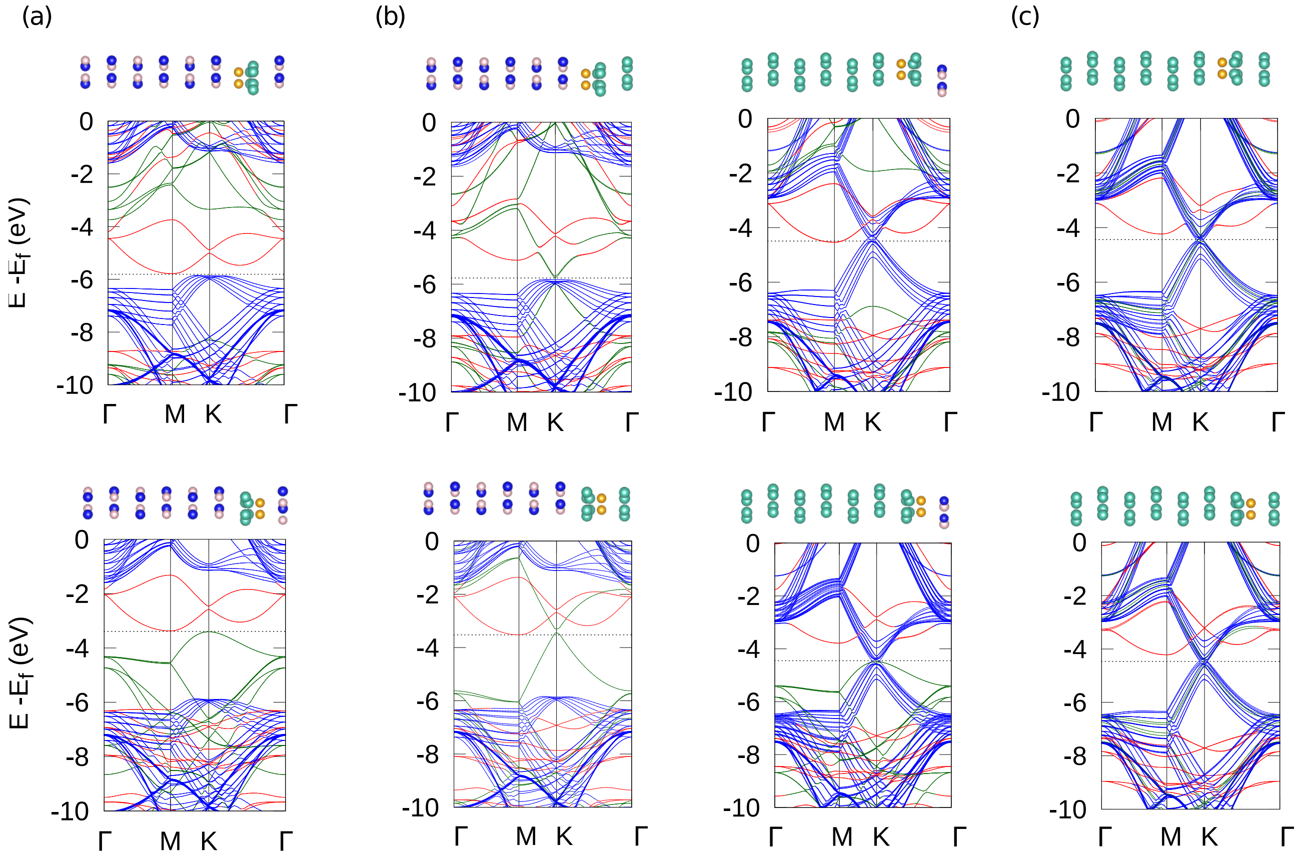

Figure S1: Band structure of CL, C_4F, and substrates. The band of substrate, C_4F, and CL is colored with blue, red, and green, respectively.

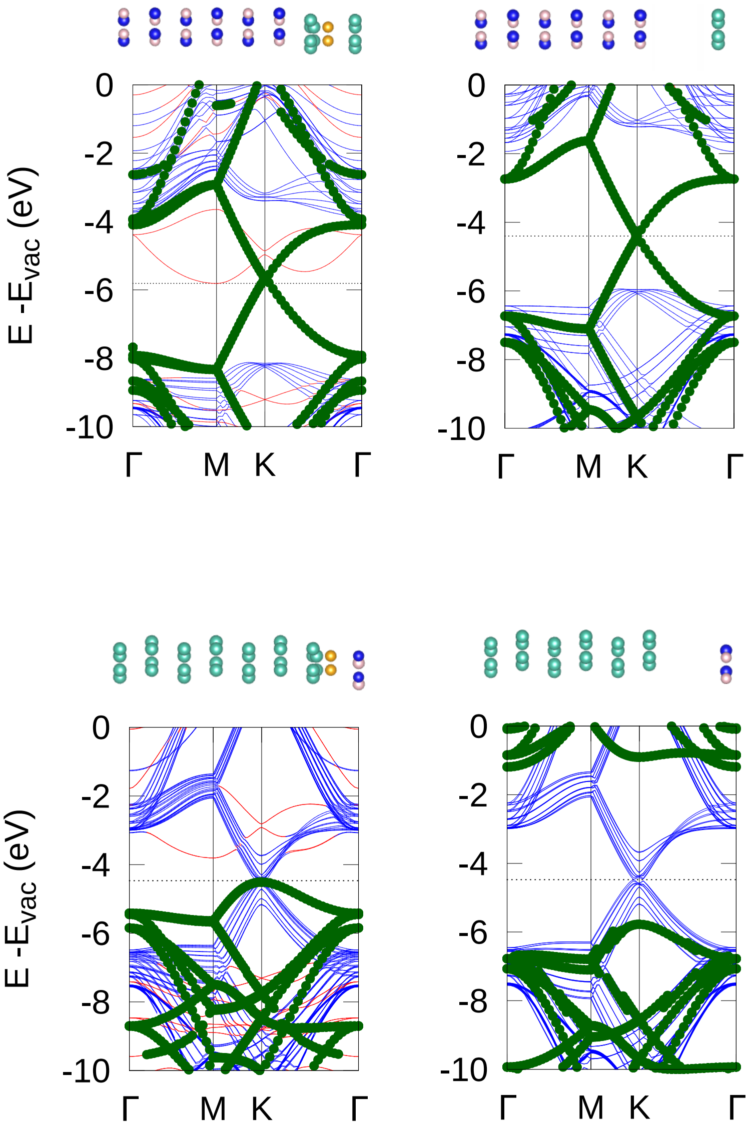

Figure S2: The band structure of CL/C_4F/substrates for type-B alignment. (a) shows the band structure of G/FC_4F/*h*-BN and G/*h*-BN. The band shift of graphene is 1.41 eV. (b) is for BN/FC_4/Grpt and BN/Grpt and the shift of BN is 1.30 eV.

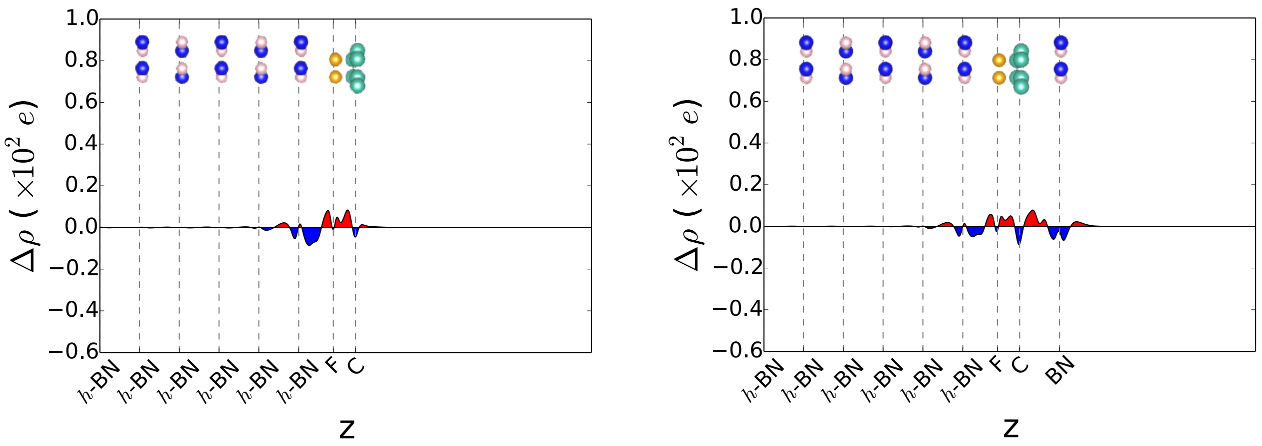

Figure S3: Electron redistribution function of (a) C_4F/BN and (b) BN/C_4F/BN. The magnitude of the redistribution is similar between two systems.
